# When, Where and How Osteoporosis-Associated Fractures Occur: An Analysis from the Global Longitudinal Study of Osteoporosis in Women (GLOW)

**DOI:** 10.1371/journal.pone.0083306

**Published:** 2013-12-11

**Authors:** Aline G. Costa, Allison Wyman, Ethel S. Siris, Nelson B. Watts, Stuart Silverman, Kenneth G. Saag, Christian Roux, Maurizio Rossini, Johannes Pfeilschifter, Jeri W. Nieves, J. Coen Netelenbos, Lyn March, Andrea Z. LaCroix, Frederick H. Hooven, Susan L. Greenspan, Stephen H. Gehlbach, Adolfo Díez-Pérez, Cyrus Cooper, Juliet E. Compston, Roland D. Chapurlat, Steven Boonen, Frederick A. Anderson, Jonathan D. Adachi, Silvano Adami

**Affiliations:** 1 Department of Medicine, Division of Endocrinology, College of Physicians and Surgeons, Columbia University, New York, United States of America; 2 Department of Medicine, Division of Endocrinology, São Paulo Federal University, São Paulo, Brazil; 3 Center for Outcomes Research, University of Massachusetts Medical School, Worcester, Massachusetts, United States of America; 4 Department of Medicine, Columbia University Medical Center, New York, United States of America; 5 Mercy Health Osteoporosis and Bone Health Services, Cincinnati, Ohio, United States of America; 6 Department of Rheumatology, Cedars-Sinai Medical Center, Los Angeles, California, United States of America; 7 Division of Clinical Immunology and Rheumatology, University of Alabama at Birmingham, Birmingham, Alabama, United States of America; 8 Paris Descartes University, Cochin Hospital, Paris, France; 9 Rheumatology Section, Department of Medicine, University of Verona, Italy,; 10 Department of Internal Medicine III, Alfried Krupp Krankenhaus, Essen, Germany; 11 Helen Hayes Hospital and Columbia University, West Haverstraw, New York, United States of America; 12 Department of Endocrinology, VU University Medical Center, Amsterdam, The Netherlands; 13 University of Sydney Institute of Bone and Joint Research and Department of Rheumatology, Royal North Shore Hospital, Australia; 14 Fred Hutchinson Cancer Research Center, Seattle, Washington, United States of America; 15 University of Pittsburgh, Pittsburgh, Pennsylvania, United States of America; 16 Hospital del Mar-IMIM-Autonomous University of Barcelona, Barcelona, Spain; 17 RETICEF, ISCIII Madrid; Spain; 18 MRC Lifecourse Epidemiology Unit, University of Southampton, Southampton, United Kingdom; 19 Cambridge University Hospitals NHS Foundation Trust, Cambridge, United Kingdom; 20 Division of Rheumatology, INSERM UMR 1033, Université de Lyon, Hospices Civils de Lyon, Hôpital E Herriot, Lyon, France; 21 Universiteit Leuven, Leuven, Belgium; 22 St Joseph's Healthcare, McMaster University, Hamilton, Ontario, Canada; 23 Department of Rheumatology, University of Verona, Ospedale, Verona, Valeggio, Italy; University of Southampton, United Kingdom

## Abstract

**Objective:**

To examine when, where and how fractures occur in postmenopausal women.

**Methods:**

We analyzed data from the Global Longitudinal Study of Osteoporosis in Women (GLOW), including women aged ≥55 years from the United States of America, Canada, Australia and seven European countries. Women completed questionnaires including fracture data at baseline and years 1, 2 and 3.

**Results:**

Among 60,393 postmenopausal women, 4122 incident fractures were reported (86% non-hip, non-vertebral [NHNV], 8% presumably clinical vertebral and 6% hip). Hip fractures were more likely to occur in spring, with little seasonal variation for NHNV or spine fractures. Hip fractures occurred equally inside or outside the home, whereas 65% of NHNV fractures occurred outside and 61% of vertebral fractures occurred inside the home. Falls preceded 68–86% of NHNV and 68–83% of hip fractures among women aged ≤64 to ≥85 years, increasing with age. About 45% of vertebral fractures were associated with falls in all age groups except those ≥85 years, when only 24% occurred after falling.

**Conclusion:**

In this multi-national cohort, fractures occurred throughout the year, with only hip fracture having a seasonal variation, with a higher proportion in spring. Hip fractures occurred equally within and outside the home, spine fractures more often in the home, and NHNV fractures outside the home. Falls were a proximate cause of most hip and NHNV fractures. Postmenopausal women at risk for fracture need counseling about reducing potentially modifiable fracture risk factors, particularly falls both inside and outside the home and during all seasons of the year.

## Introduction

Osteoporosis is a disease in which age-related reductions in bone strength predispose to low-trauma fractures. Worldwide, several million post-menopausal women experience such fractures each year, with attendant pain and disability, temporary or permanent reductions in quality of life, and substantial economic costs borne personally and by healthcare systems [1], [2]. Efforts are being made to identify those at increased risk of fracture, to optimize calcium and vitamin D use, encourage physical activity when possible and prescribe effective anti-osteoporosis treatments as appropriate. To enhance further our ability to counsel women about fracture risk as they age, we believed that it would be useful to examine the proximate causes of low-trauma fractures by ascertaining when during the year women sustain their fractures, where the events that lead to a fracture event occur, and how different fractures typically happen.

We evaluated data obtained from the international Global Longitudinal Study of Osteoporosis in Women (GLOW) to characterize the circumstances surrounding incident low-trauma fractures in a primary care population of postmenopausal women aged 55 years and older from the United States of America, Canada, Australia and several countries in northern and southern Europe. In this paper, we offer a descriptive analysis of when, where and how vertebral, hip, and non-hip, non-vertebral (NHNV) fractures occurred during the first 3 years of observation of the GLOW cohort.

## Methods

### Ethics Statement

Each study site obtained ethics committee approval to conduct the study in the specific location.

### Study Design

GLOW is a longitudinal database study being conducted in physician practices at 17 sites in 10 countries (Australia, Belgium, Canada, France, Germany, Italy, The Netherlands, Spain, United Kingdom and United States of America). These sites are located in major population centers. Details of the study design and methods have previously been described [3]. Study sites were selected to achieve a broad geographic distribution across several countries in northern and southern Europe and within the US, as well as to have participation from both Canada and Australia. All study sites had lead investigators with expertise in osteoporosis and access to a clinical research team capable of managing a large cohort of subjects. These lead investigators identified primary care practices in their region that were members of local research or administrative networks and able to supply names and addresses of their patients electronically. The composition of groups varied by region and included health-system owned practices, managed practices, independent practice associations and health maintenance organizations. Networks established for the purpose of general medical research were used only if they were not established exclusively for osteoporosis research and did not consist primarily of physicians whose primary focus was academic.

Primary care physicians were defined as doctors who spent most of their time providing primary healthcare to patients, and included internists, family practitioners and general practitioners. Each practice provided a list of the names and addresses of women aged 55 years and older who had been attended by their physician in the past 24 months. Sampling was stratified by age to ensure that two-thirds were women aged 65 years and older. Patients were excluded if they were unable to complete the study survey due to cognitive impairment, language barriers, institutionalization or illness.

### Questionnaire Development

Questionnaires were designed to be self-administered and the baseline instrument covered domains that included: patient characteristics and fracture risk factors, patients' perceptions about fracture risk and osteoporosis, past or current use of medications (including osteoporosis treatment), diagnosed comorbidities, healthcare use and access, physical activity, physical function and quality of life. Where possible, items from published validated instruments were used, including the National Health and Nutrition Examination Survey [4], the EQ-5D [5] and the SF-36 [6] (physical function and vitality components). Questions that had not been used previously were tested cognitively in the context of the complete questionnaire in a sample of women in the study age group. Questionnaires were translated into five languages (French, Spanish, German, Italian and Dutch) in addition to English by the University of Massachusetts-Amherst Translation Center.

Baseline questionnaires, along with invitations to participate in the study signed by the local principal investigator, were mailed to all potential subjects. Non-respondents were followed up with sequential postcard reminders, second questionnaires and telephone interviews. Questionnaires were mailed at 1, 2 and 3 years to collect information about incident fractures. Questions were designed to determine which bone or bones were fractured, if the fracture occurred inside or outside the home, how the fracture happened, and during what time of year the fracture event occurred.

### Statistical Analysis

Fracture rates during the first 3 years of follow-up are reported as raw numbers and percentages. Differences were tested using the chi-square test or Fisher's exact test in the case of small cell values; the Mantel-Haenszel chi-square test was used when comparing ordered age categories. Data are based upon individual subjects and not individual fractures, and reflect each woman's first incident fracture post baseline. Women with missing fracture data were counted as not fracturing. We have separated the fractures into three types: hip, vertebral and NHNV (defined as fracture of the clavicle, upper arm, wrist, rib, pelvis, ankle, upper leg, lower leg, shoulder, knee, hand, elbow or foot). If a woman had more than one fracture type in one year, she was excluded from the analysis. Vertebral fractures were assumed to be clinical vertebral fractures predominantly, rather than morphometric vertebral fractures, as these were reported based upon self-report after a clinical diagnosis by a physician.

The possible answers concerning how the fracture happened were: “caused by a fall” (fell on stairs; fell climbing on a chair, stool or ladder; fell out of bed or off a chair; slipped or tripped); “caused by a severe trauma” (sporting injury; motor vehicle accident; heavy object fell or hit the body); “bone broke with no fall or injury”; and “other” when it was marked as the answer. In the year 3 survey only, two additional response choices were added: “fainted or lost consciousness” and “my legs gave way”. These were included in the category of “caused by a fall”.

The calendar season of the fracture was determined by the month in which it was reported: in all sites except Australia, the months of December, January and February were considered winter; March, April and May were considered spring; June, July and August were considered summer; and September, October and November were considered fall. In Australia, the seasons were reversed.

The data were analyzed for all subjects combined, and are also described for four age subgroups: ≤64, 65–74, 75–84 and ≥85 years for the analysis of how fractures occurred. All analyses were conducted using SAS version 9.2.

## Results

Between October 2006 and February 2008, a total of 60,393 postmenopausal women were enrolled across the 10 participant countries, including those from practices in the United States of America (28,170), France (5080), the United Kingdom (4079), Canada (3985), Belgium (3692), Germany (3465), Italy (3252), Australia (2904), Spain (2910) and the Netherlands (2856). Of the 60,393 women enrolled, 51,491 completed the year 1 questionnaire, 48,750 completed the year 2 questionnaire and 45,490 completed the year 3 questionnaire; there were 42,216 women with continuous follow-up for all survey years for a crude total response rate of 70%.

A total of 4268 first incident fractures were reported during the first 3 years of follow-up. Women who reported multiple fractures (n = 146) were excluded from the analysis, leaving a total of 4122 first incident single fractures. Basic demographics for all groups are shown in Table 1. The majority were NHNV fractures (n = 3542, 86%), followed by vertebral (n = 349, 8%) and hip (n = 231, 6%) fractures.

**Table 1:** Baseline characteristics, overall and by fracture type (n = 4,122).

	Any Fracture (n = 4,122)	Type of Fracture			Excluded (n = 146)
		Hip (n = 231)	Spine (n = 349)	NHNV (n = 3,542)	
Age	69 (62–77)	77 (71–83)	72 (66–79)	68 (62–76)	74 (65–82)
Body mass index	26 (23–29)	25 (22–28)	25 (23–29)	26 (23–29)	26 (22–29)
Region					
Canada/Australia	502 (12)	17 (7.4)	36 (10)	449 (13)	25 (17)
Europe	1,645 (40)	90 (39)	126 (36)	1,429 (40)	57 (39)
USA	1,975 (48)	124 (54)	187 (54)	1,664 (47)	64 (44)
Number of co-occurring conditions*					
0	1,386 (34)	59 (26)	99 (28)	1,228 (35)	30 (21)
1	1,704 (41)	106 (46)	139 (40)	1,459 (41)	57 (39)
≥2	1,032 (25)	66 (29)	111 (32)	855 (24)	59 (40)
Number of falls in past 12 months					
0	2,104 (52)	113 (50)	177 (51)	1,814 (52)	61 (42)
1	1,039 (26)	55 (25)	95 (27)	889 (25)	38 (26)
≥2	923 (23)	56 (25)	75 (22)	792 (23)	46 (32)
Prior fracture at baseline	1,527 (38)	87 (39)	158 (46)	1,282 (37)	86 (60)
Weight <125 lb (<57 kg)	721 (18)	54 (24)	62 (18)	605 (17)	30 (21)
Parental history of hip fracture	760 (21)	47 (23)	70 (23)	643 (21)	29 (25)
Current smoker	374 (9.2)	13 (5.7)	40 (12)	321 (9.2)	12 (8.4)
Current glucocorticoid	194 (4.8)	13 (5.7)	26 (7.6)	155 (4.5)	14 (9.9)
Alcohol misuse (>20 drinks/week)	28 (0.7)	2 (0.9)	0 (0.0)	26 (0.7)	1 (0.7)

%). Data given as median (interquartile range) or count (

Asthma, chronic bronchitis or emphysema, osteoarthritis or degenerative joint disease, rheumatoid arthritis, stroke, ulcerative colitis or Crohn's disease, celiac disease, Parkinson's disease, multiple sclerosis, cancer, type 1 diabetes.

–non-vertebral. NHNV, non-hip

### When Fractures Occurred

**Figure 1:**
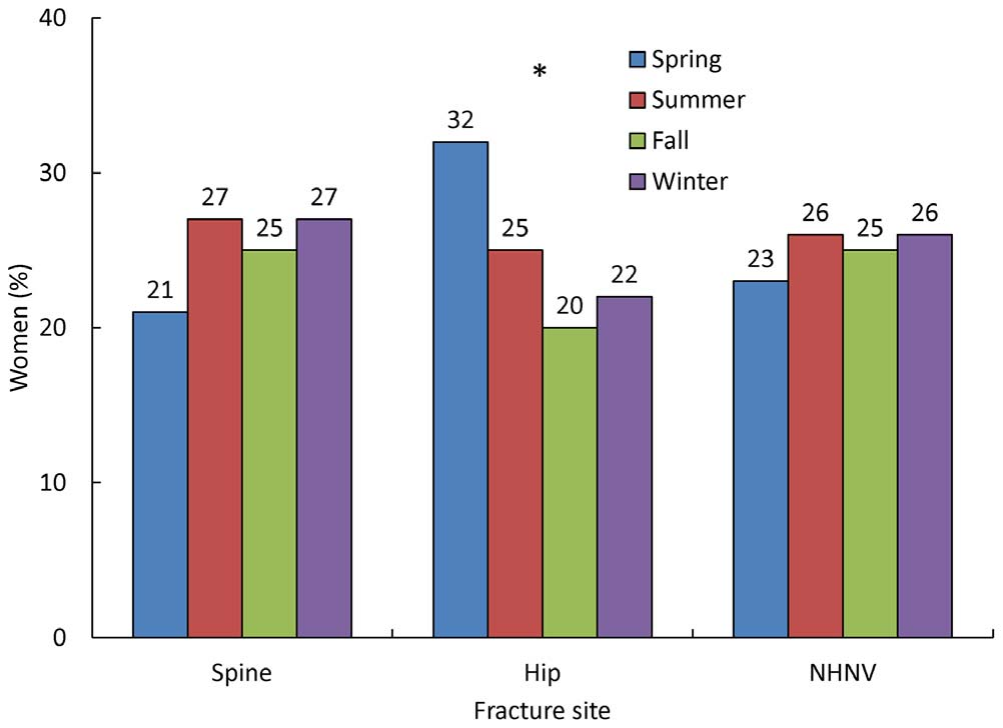
Seasonal variation of fracture rates according to fracture site. Significant differences for each season within fracture types (*P*<0.05) are denoted by an asterisk. NHNV, non-hip, non-vertebral.

As shown in Figure 1, fractures in the GLOW population occurred across the seasons of the year. For spine and NHNV fractures, the small seasonal variability was not significant. However, there was significant variation in the rate of hip fractures across the seasons (*P* = 0.01), with a significantly higher hip fracture rate in the spring (32% occurred then) compared with the other seasons. Looking at the United States of America only, the seasonal pattern of hip fractures was similar, but was only borderline significant (*P* = 0.05).

### Where Fractures Occurred

**Figure 2:**
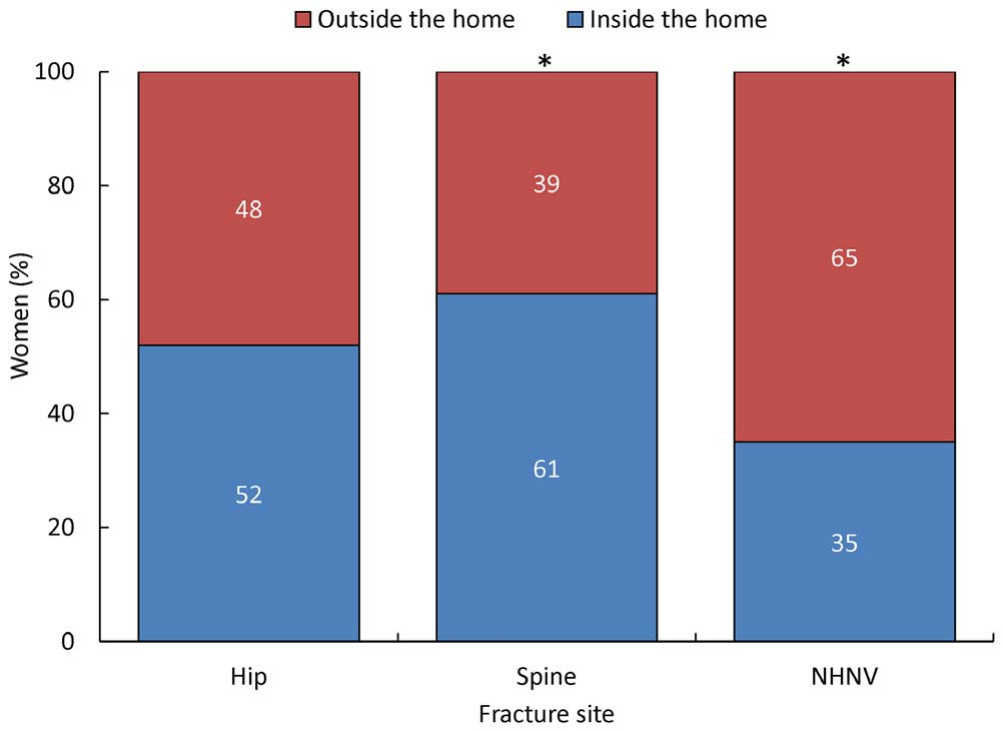
Location of occurrence of fracture by fracture type. Significant differences for each location within fracture types (*P*<0.05) are denoted by an asterisk. NHNV, non-hip, non-vertebral.

Figure 2 illustrates the location of the fracture event by fracture type. Fractures were described as occurring inside or outside the home. For hip fractures, there was a fairly even division between inside and outside (52% vs 48%, *P* = 0.45). Significantly more vertebral fractures occurred inside the home than outside (61% vs 39%, *P*<0.001), while NHNV fractures occurred more commonly outside than in the home (65% vs 35%, *P*<0.001).

### How Fractures Occurred

**Figure 3:**
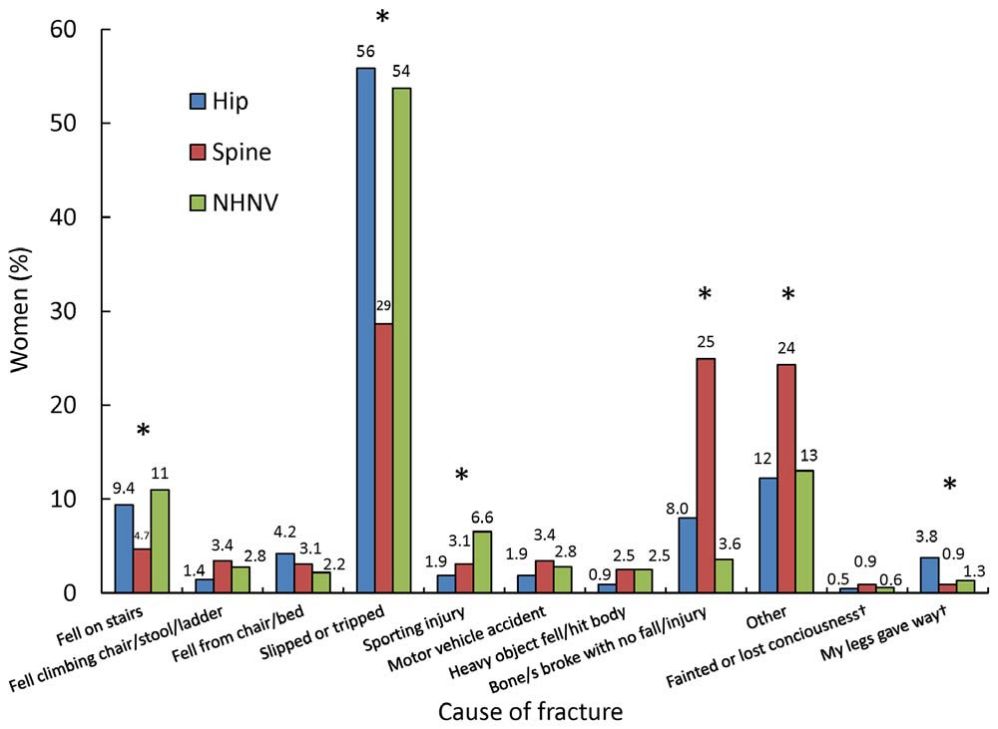
Cause of fracture by fracture type. Significant differences for each cause between fracture types (*P*<0.05) are denoted by an asterisk. Dagger denotes response only available on year 3 survey. NHNV, non-hip, non-vertebral.

The events leading to a fracture are shown in Figure 3, depicting the percentages of women reporting the various causes of fracture by each of the three fracture types. Overall, the most common proximate cause for any fracture type was slipping or tripping (more than half of hip and NHNV fractures and almost 30% of spine fractures). The proportions of spine fractures due to slipping/tripping or from a fall on the stairs were significantly lower than for the other two types of fracture (*P*<0.05). A relatively small proportion of women experienced fractures related to sporting injuries, but this category accounted for a significantly higher proportion of NHNV fractures compared to the other two fracture types (*P*<0.05). Fractures with no fall or injury and “other” reasons accounted for a significantly greater proportion of spine fractures than of the other two fracture types (*P*<0.05).

**Figure 4:**
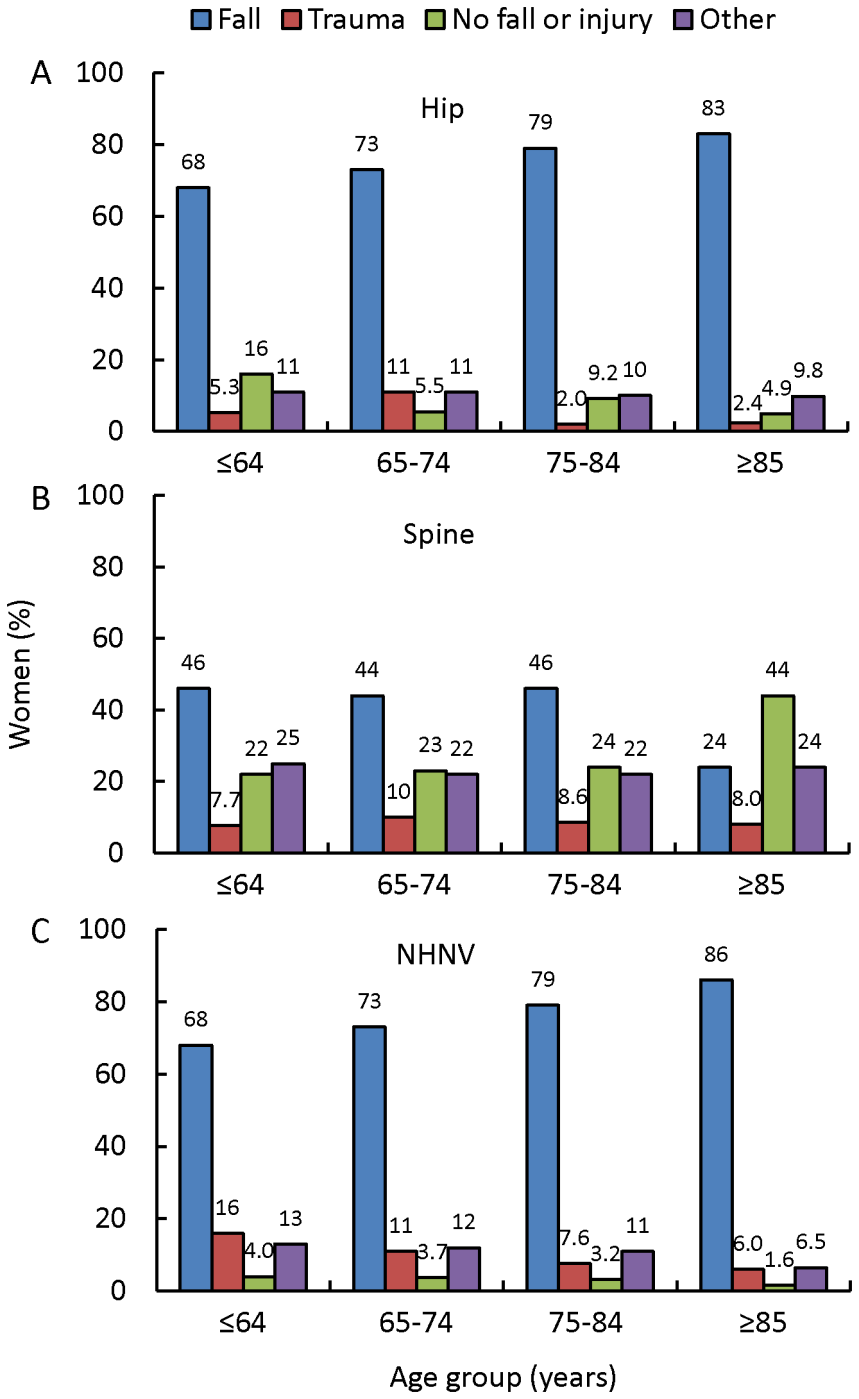
Cause of fractures by age group. A, For hip fractures. B, For spine fractures. C, For NHNV fractures. NHNV, non-hip, non-vertebral.

Circumstances associated with fractures by age group are shown in Figure 4. Of note, a fall as the proximate cause of hip fracture rose from about two thirds of cases in women aged ≤64 years and trended upward (*P* = 0.13) with advancing age to being the cause of more than 80% of hip fractures in the most elderly group (Figure 4A). This trend was likely not significant due to the small number of hip fractures leading to a lack of power to show a significant relationship. Conversely, trauma as the cause of hip fracture, which accounted for about 10% in women aged 65–74 years, decreased to approximately 2% in women aged ≥75 years. A small minority of hip fractures occurred with no fall or injury in all age groups, and as age increased the absence of a fall or injury at the time of hip fracture became even less common. For spine fractures, about 45% were associated with a fall and about 20% were associated with no fall or injury in all age groups except those aged ≥85 years, for whom these findings were reversed, with falls accounting for 24% and no fall or injury for over 40% in this oldest group (Figure 4B). For NHNV fractures, there was a significant upward trend (*P*<0.0001) for a fall as the proximate cause with increasing age (and a consistent significant downward trend (*P*<0.0001) for trauma for each older age grouping ) (Figure 4C).

## Discussion

To determine when, where and how hip, spine and NHNV fractures occur in postmenopausal women, we analyzed data from the first 3 years of GLOW. In this large, international, population-based study of women aged 55 years and older, there were 4122 self-reported first incident fractures, of which most (86%) were NHNV fractures. Hip fractures occurred nearly equally inside and outside the home, while most spine fractures occurred inside the home and most NHNV outside the home. Falls were the dominant cause for hip and NHNV fractures, and the proportion of these fractures with a fall as the proximate cause increased with increasing age.

The effect of seasonality on fractures, especially hip fractures, has previously been reported from large series in Scandinavia, the United States of America and Canada. Recent studies from different areas of Norway have found that hip fracture rates are highest during the winter [7], particularly for those fractures occurring outdoors [8], while two older studies did not see a seasonal variation of effects of cold temperature on the incidence of hip fractures in Oslo, Norway [9] or Malmo, Sweden [10]. Two Medicare-based population studies conducted in the United States of America in the 1980s found that hip fracture rates [11] and fracture rates for hip, distal forearm, proximal humerus and ankle fractures [12] were highest in the winter and lowest in the summer. A study from Canada with data from 1982–1992 found hip fractures in women aged 60–74 years peaked in mid-December [13]. The prospective data from GLOW show similar proportions of NHNV fractures throughout the four seasons, and a significantly higher number of hip fractures during spring compared with the other three seasons. It is possible that lower levels of vitamin D resulting from a reduction in sunlight exposure over the winter and clinically manifest in spring might be a contributing factor to the occurrence of hip fracture in GLOW subjects, but there were no collections of 25-hydroxy vitamin D data in GLOW to support or refute this possibility. It is not clear why these data differ from those in previous studies, but our results may reflect the broad geographical distribution of the GLOW countries with a variety of climates, or may represent secular changes in the timing of fractures since the mid-to-late 1980s, when data for many of the previous studies were collected.

In GLOW, almost twice as many NHNV fractures occurred outside the home as inside the home, a finding consistent with previous studies that have noted that the majority of NHNV fractures occur outdoors [14], [15]. However, we found almost no difference in the number of hip fracture events occurring inside or outside the home, in contrast with earlier reports that indicate that most hip fractures originate at home or in hospices, particularly if caused by a fall [14]-[17]. The GLOW population consists of ambulatory women who may be more health aware and active than those in earlier studies, particularly as they were recruited from the practices of the primary care physicians whom they routinely visited and had volunteered to be a part of this study.

This study confirms the importance of falling as the principal proximate cause for the majority of hip and NHNV fractures, increasing in frequency as a cause of fracture with advancing age. Though almost half of vertebral fractures in postmenopausal women aged under 85 years were also preceded by some type of fall, in the most elderly (>85 years), vertebral fractures were most often reported in the absence of any fall or injury. While fall prevention strategies have not been proven to lower fracture rates, it may still be desirable for patients at risk for fractures to be provided with advice on how to lower the risk of falls, and efforts to reduce falls both in the home and outside the home should be applied throughout all seasons of the year, not solely in the winter.

A major strength of this study is the use of a standard questionnaire for all countries, minimizing methodology bias by collecting data in a uniform manner. Owing to the very large sample with a broad age range, GLOW provides us with information representative of women at risk of osteoporosis-related fractures, including women younger than 65 years, as well as those who are over 85 years of age.

This study has several important limitations. Fracture information was self-reported. Previous studies have shown that self-report is a reliable and accurate method to obtain information of previous major fractures, with a greater ascertainment for hip and distal forearm fractures, but less accurate for clinical vertebral fractures [18]-[20]. Vertebral fractures are often asymptomatic, or at least more likely to be clinically unrecognized, which may account for the lower accuracy of self-report information, and the underestimation of their incidence [21], [22]. In GLOW, we assumed most vertebral fractures reported by the women were clinical, but it is possible that some were morphometric fractures noted on a radiograph taken for some other reason following which women were told there was evidence of a fracture not previously recognized clinically. Women with multiple fractures were excluded from the analysis, and those women tended to be older and in worse health. However, given the relatively small number of excluded women their removal is unlikely to significantly alter the findings of this paper. Finally, vital status was not collected so death rates and adjusted survey response rates cannot be calculated.

## Conclusions

GLOW is the first multi-national study to provide a broad and detailed overview of when, where and how contemporary postmenopausal women experience fractures in several regions of the United States of America, in parts of several countries in both northern and southern Europe, in eastern Canada and in southern Australia. The information provided in this report underscores the importance of teaching older women who may be at risk for fracture about falling as an important event that leads to fracture as well as to emphasize that fractures occur in all seasons of the year as well as within and outside the home. Such information should offer clinicians the opportunity to enhance patient awareness about these aspects of fracture risk that may be helpful to them.
